# Epidemiology of Herpes Simplex and Varicella Zoster Virus-Associated Central Nervous System Infections in Western Greece: A Five-Year Retrospective Analysis

**DOI:** 10.3390/pathogens15010030

**Published:** 2025-12-24

**Authors:** Vasileios Kakouris, Niki Kalyva, Maria Militsopoulou, Vassiliki Stamouli, Georgios Meletis, Melina Kachrimanidou, Fotini Paliogianni

**Affiliations:** 1 Department of Microbiology, University General Hospital of Patras, Rio, 26504 Patras, Greece; vaskakouris@gmail.com (V.K.); niki.kalyva0612@gmail.com (N.K.); mariamilitsopoulou@yahoo.com (M.M.); vstam@pgnp.gr (V.S.); 2 Department of Microbiology, Medical School, Aristotle University of Thessaloniki, 54124 Thessaloniki, Greece; gmeletis@auth.gr (G.M.); melinaka@auth.gr (M.K.)

**Keywords:** CNS, herpes viruses, HSV-1, HSV-2, VZV

## Abstract

The epidemiology of central nervous system (CNS) infections caused by herpesviruses varies with host factors and geographic distribution. Timely diagnosis and therapeutic intervention are life-saving. This study investigated the epidemiology of herpesvirus CNS infections in Western Greece, compared clinical and laboratory findings with international data and evaluated an internal laboratory algorithm for cerebrospinal fluid (CSF) molecular testing criteria. During the study period, 940 of 4300 CSF samples met eligibility criteria for RT-PCR detection of *herpes simplex virus* (HSV-1, HSV-2) and varicella zoster virus (VZV). Of these, 53 (5.63%) were positive: 37 VZV, 9 HSV-1, and 7 HSV-2. HSV-2 cases occurred in younger patients (median age 41) and had the highest CSF white blood cells (WBC) counts (231/mm^3^), followed by VZV (125/mm^3^) and HSV-1 (26/mm^3^). CSF protein was higher in HSV-2 infections. Magnetic resonance imaging (MRI) was the most sensitive imaging modality for detecting CNS inflammation. These results indicate VZV as the predominant herpesvirus in this region, underscoring the need for high clinical suspicion in older patients and timely molecular diagnosis.

## 1. Introduction

Alpha-herpesviruses, comprising *herpes simplex viruses* 1 and 2 (HSV-1, HSV-2) and Varicella Zoster virus (VZV), are double-stranded DNA viruses with marked neurotropism [1]. They cause recurrent infections with low mortality, except in cases involving central nervous system (CNS) infection, where outcomes depend on the etiologic agent and host immune status [1,2].

CNS infections (meningitis and encephalitis) are increasing due to the growing population of hospitalized immunocompromised and elderly patients [2]. The introduction of targeted vaccination programs has substantially reduced the incidence of alpha-herpesvirus-associated central nervous system infections, particularly those caused by VZV. Widespread immunization against bacterial infections in infancy has altered the epidemiology of CNS infections by decreasing primary disease and associated neurological complications. A shift from bacterial to viral meningitis highlights the success of vaccination programs and underscores the continuing clinical significance of viral CNS infections across all age groups. Nevertheless, continued surveillance remains essential, as shifts in population immunity and viral reactivation patterns may influence the residual burden of CNS disease [3]. Most current cases of aseptic meningitis or encephalitis are of viral origin, with enteroviruses responsible for over 80% of viral meningitis [3,4,5]. Viral meningitis generally has a favorable prognosis and is managed supportively, except for herpesvirus infections, which require specific antiviral therapy [6,7,8]. Because clinical manifestations overlap across etiologies, bacterial meningitis must always be considered, as prompt diagnosis and treatment are essential to reduce morbidity and mortality [5,9]. The annual incidence of meningitis is approximately 13.5 per 100,000, with viral meningitis accounting for about 2.75 per 100,000 in the United Kingdom, varying by age, time, and region [3,7,9,10]. Geographic and environmental factors, including population movement and climate change, contribute to localized outbreaks and emerging pathogens [4,11].

Encephalitis is the second most significant CNS infection and, along with meningitis, constitutes a neurological emergency. Depending on lesion localization, it may present with seizures, altered consciousness, behavioral changes, or focal neurological deficits, including cranial nerve involvement [9]. In most cases, the etiology remains unidentified, though viruses predominate [11]. Herpesviruses, particularly HSV-1, are the leading causes, with HSV-1 responsible for severe, potentially fatal encephalitis if untreated, followed by VZV [12,13]. The annual incidence of sporadic infectious encephalitis ranges from 1.5 to 7 cases per 100,000 population, varying with age [7,13].

The incorporation of polymerase chain reaction (PCR) into diagnostic workflows has markedly enhanced the detection of viral CNS infections, allowing rapid identification of pathogens in cerebrospinal fluid with high sensitivity and specificity (96–98% and 95–99%, respectively) [12,14,15,16,17,18,19,20]. Despite these advances, a substantial proportion of cases remain of unknown etiology. The broader application of PCR has also highlighted an increasing incidence of alpha-herpesvirus-associated CNS infections, warranting their routine consideration in all suspected cases [9,12,14,19].

This study aimed to investigate the epidemiology of alpha-herpesvirus-associated CNS infections in Western Greece and compare the findings with data from other regions. Demographic, laboratory, and imaging characteristics were analyzed by viral subtype to assess geographic variations. Additionally, the performance of an internal algorithm integrated into the molecular diagnostic workflow for herpesvirus detection was evaluated.

## 2. Materials and Methods

### 2.1. Data Acquisition-Characteristics of Population

This retrospective study analyzed clinical and laboratory data from inpatients and outpatients at the University General Hospital of Patras from 1 January 2019 to 30 June 2024. All cerebrospinal fluid (CSF) samples obtained via lumbar puncture underwent cell count, differential, biochemical analysis (glucose, protein), Gram and Ziehl–Neelsen staining, and culture in enriched media. The cell differential of CSF was determined by light microscopy followed by May–Grünwald and Giemsa staining. Herpesvirus (HSV-1, HSV-2, VZV) screening followed a laboratory algorithm based on prior experience and literature, targeting samples with ≥10 cells/mm^3^ and/or protein ≥40 mg/dL [16,20,21,22]. However, in coordination with treating clinicians, testing was also performed in cases with high clinical suspicion, even if the initial laboratory criteria were not fully met, particularly in immunocompromised patients. Exclusion criteria included CSF samples from patients with an alternative clinical or laboratory diagnosis (e.g., CSF collected for multiple sclerosis monitoring or diagnosis), post-surgical CSF, or CSF obtained from shunts. Only one CSF sample per patient was included, even if repeat lumbar punctures were performed for ongoing clinical suspicion. As shown by the analysis of the cases, none of the patients had COVID-19 at the time of diagnosis or during hospitalization. Evaluated laboratory parameters included inflammatory markers (C-reactive protein), complete blood count (CBC) with differential, immunoglobulins, biochemical profile, and serology for other pathogens. Demographic characteristics were not exclusion criteria.

### 2.2. Description of PCR

All CSF samples were stored at 4 °C until the day of testing (3–5 days). The test involved a multiplex real-time amplification reaction using an automated, integrated system for nucleic acid extraction, real-time amplification, and result interpretation (Real-Time PCR, ELITE InGenius, ELITECH GROUP, Puteaux, France).

Following DNA extraction from the tested samples, three specific amplification reactions were performed in special tubes, each targeting the following viruses:

1. **HSV-1 (*Herpes Simplex Virus* Type 1):** Detected by a specific probe targeting the gene encoding glycoprotein G.
2. **HSV-2 (*Herpes Simplex Virus* Type 2):** Detected by a specific probe targeting the gene encoding glycoprotein D.
3. **VZV (Varicella *Zoster Virus*):** Detected by specific probes targeting the UL21 and ORF62 genes.

Nucleic acids were extracted from patient samples in parallel with internal control (IC) to assess the efficiency of the extraction process and to detect potential PCR inhibition by sample factors.

The IC was based on an exogenous target—specifically, murine cytomegalovirus (MCMV) sequences—detected using a dedicated probe.

This qualitative method has a clinical sensitivity >97% for HSV-1 and 100% for HSV-2 and VZV, with 100% specificity for all three viruses [23,24].

### 2.3. Statistical Analysis

The statistical analysis of the findings was performed using the SPSS software package, version 28.0. The Chi-Square test and the Kruskal–Wallis test were used, as appropriate, to identify differences in parameters between the viruses. A *p*-value of <0.05 was considered statistically significant.

## 3. Results

During the 5-year study period in the University Hospital of Patras, 4300 CSFs were received and tested in the laboratory. Only 940 fulfilled the clinical and laboratory eligibility criteria for further molecular testing. Samples that were excluded either did not meet the eligibility criteria or another microbial agent was identified with a different laboratory method.

### 3.1. HSV-1, HSV-2 and VZV Positivity

Among the 940 CSFs that were tested with a molecular method, 53 of them were positive for one of the three viruses tested (5.6%). 37/53 (69.8%) were identified with VZV, while 9/53 (17%) and 7/53 (13.2%) were positive for HSV-1 and HSV-2, respectively. None of these three a-herpesviruses was isolated in the rest of the 887 CSFs.

### 3.2. Demographic Characteristics of Patients Infected with HSV-1, HSV-2 or VZV

Positive samples for one of the tested viruses were detected in 30 males and 23 females correspondingly. The gender distribution per virus did not reveal a statistically significant difference between the two sexes (*p* = 0.46).

**Figure 1:**
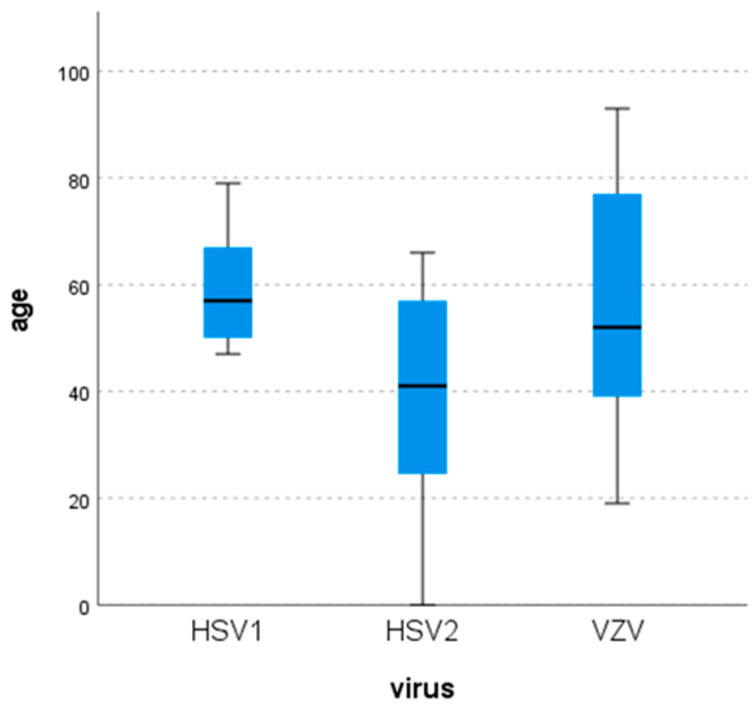
Age distribution of positive cases for the tested viruses.

Age distribution did not vary significantly in different ages among different viruses (*p* = 0.185). Specifically, HSV-1 was detected in patients between 50–80 years of age, while HSV-2 had a wider distribution from a newborn to a 66-year old patient. Compared with the HSV-2 group, the median age in the HSV-1 group was older [for HSV-1: 57 years (47–79); for HSV-2: 41 years (0–66)]. Concerning patients with VZV infection, the median age was 52 years while the age distribution was wide (19–93) even though 50% of the cases were between 39–78 years (Figure 1). Moreover, the maximum and the upper quartile values were higher than those in the other two groups.

### 3.3. Seasonal Distribution

No statistical differences were detected among different viruses detected in different times of year, but it was prominent that most HSV-1 and HSV-2 cases occur during the autumn and winter months, whereas VZV cases are distributed throughout the entire year.

Also, a similar distribution of cases was noted for all viruses tested throughout the whole study period. It should be noted that cases from 2024 were recorded up to 30 June 2024 (Table 1).

### 3.4. Clinical Findings

Although a comprehensive review of clinical data was not possible due to lack of systematic recording, most cases presented with fever, headache, vomiting and neck stiffness. In cases involving inflammation of the brain parenchyma, symptoms such as confusion, focal neurological signs, and seizures were reported. Rashes and pre-existing traumatic brain injuries were reported less frequently. The immunological status in almost half of the positive cases was not available. We summarize available data in the following table (Table 2).

### 3.5. Laboratory Findings

**Figure 2:**
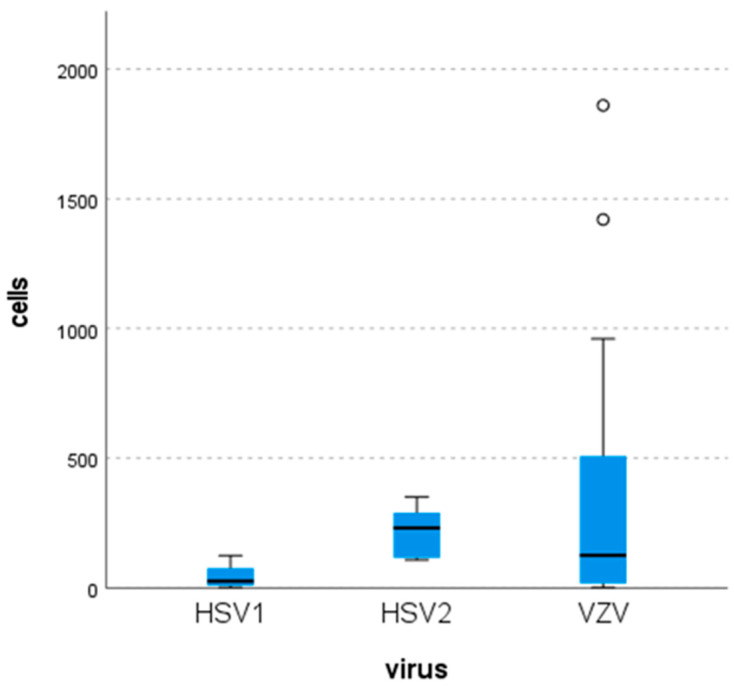
Distribution of white blood cell counts in the cerebrospinal fluid (CSF) by virus and their corresponding range.

CSF cell counts were evaluated for all samples. The mean cell count for HSV-1 was 26 cells/mm^3^ (range: 1–124), which was significantly lower compared to HSV-2, 231 cells/mm^3^ (range: 107–350), and VZV, 125 cells/mm^3^ (range: 1–1860) (Figure 2). These differences were found to be statistically significant (*p* = 0.049). Differential cell count analysis revealed a lymphocytic predominance in cerebrospinal fluid in all 53 positive cases. Monocytes represented the second most frequent cell population.

**Figure 3:**
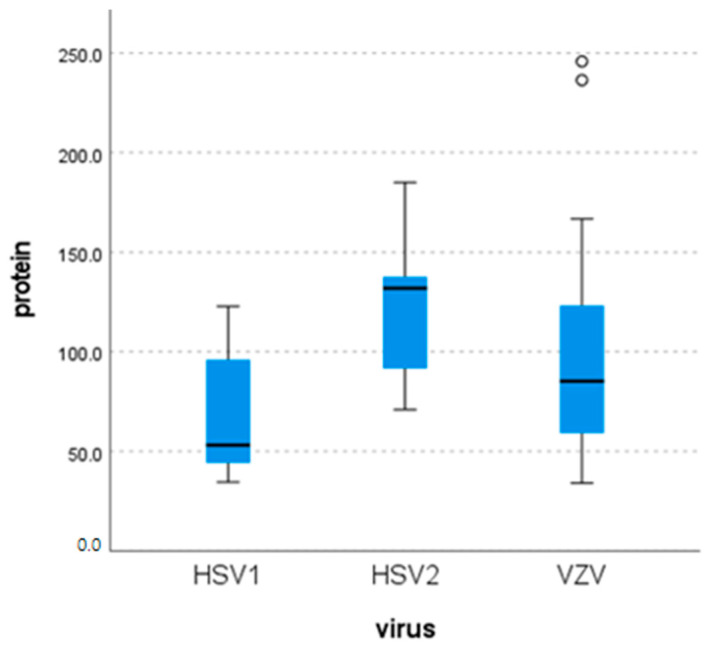
Distribution of CSF protein detected in different viruses.

Biochemicals were available in 45 cases. CSF protein reached the highest level in HSV-2 cases with a mean value of 131.8 mg/dL (range: 71–185), whereas in HSV-1 and VZV cases, lower protein levels were detected (Figure 3). The detected differences were not statistically significant (*p* = 0.051).

CSF glucose levels in all cases were similar to approximately two-thirds of the serum glucose value.

### 3.6. Cranial Imaging

Imaging data from positive cases included magnetic resonance imaging (MRI) and/or computed tomography (CT) scans. Lesions detected on positive MRIs were typically observed on T2-weighted sequences, most frequently involving the temporal and frontal lobes of the brain. Pathological MRI findings were identified in 16 out of 31 cases where data were available. The distribution of positive and negative MRI findings by virus revealed that pathological MRI findings were more frequently observed in HSV-1 cases (6 out of 7 cases with HSV-1). However, no statistically significant differences were found among the findings associated with the three viruses (*p* = 0.093). We present the MRI findings in Table 3.

CT scans revealed no pathological findings across all viral cases.

Cell counts and biochemical profiles in positive samples (where available) are summarized in Table 4.

## 4. Discussion

Members of a-herpesviruses family can cause severe to life-threatening infections when they affect the CNS. Epidemiology and laboratory diagnosis change over time given the prolonged survival of immunocompromised/immunosuppressed individuals and the inclusion of molecular methods in the diagnostic armamentarium. This retrospective study analyzed demographic as well as laboratory data of patients with CNS infections caused by one of the three α-herpesviruses, regardless of disease localization. Data were obtained from cases collected during a five-year study interval.

The positivity rate for herpes viral CNS infections was 5.63% (53 of 940 CSF samples). This study could not clearly differentiate meningitis from encephalitis cases, whereas most literature examines them separately. In comparison, Lee et al. reported a 15.2% positivity rate among patients over 16 years (80 of 525 samples: 59 meningitis, 21 encephalitis) [25]. Positivity rates vary by diagnostic thresholds and clinical indications for testing.

In this study, VZV was the most frequently detected α-herpesvirus in CNS infections, consistent with several reports favoring VZV [2,6,8,14,15,25,26,27,28]. Other studies, however, identify HSV-1 as predominant [3,4,11,19,29], with HSV-2 less common [5,10]. For instance, Kadambari et al. (England, NHS data; 22,114 CSFs) found HSV-1/2 in 17.8% and VZV in 11.4% of cases [3]. McGill et al. (UK; 1126 meningitis cases) reported 52 HSV-2, 43 VZV, and 3 HSV-1 detections [10]. In Australia, VZV appeared in 53/1128 CSFs (5%) versus HSV-1 in 21/3328 (0.7%) and HSV-2 in 47/3133 (2%) [6]. Arruti et al. (Spain) found VZV in 11.4% of patients over 65, exceeding HSV rates [2]. In England, 38 of 203 encephalitis cases were HSV-positive and 10 VZV-positive [11]. An Israeli study (908 samples) found 23 VZV, 16 HSV-1, and 5 HSV-2 [15]. Geographical distribution significantly contributes to the variations observed across different studies.

HSV-2-associated CNS infections occurred at younger ages than those caused by HSV-1 and, to a lesser extent, VZV. Nevertheless, the observed difference did not achieve statistical significance (*p* = 0.185). Most studies found no significant sex or age differences [4,6,14,15,19,28,30], though one reported a female predominance in HSV-2 cases and gender-related variations in HSV-1 and VZV incidence by age [31]. A Korean study found mean ages of 58 years for HSV-1 and 38 years for HSV-2 cases [25]. A meta-analysis also noted age-related differences in HSV-1 versus HSV-2 meningitis and encephalitis, with meningitis generally occurring at younger ages [2,19,20,27,32]. Variations in sexual behavior, where HSV is primarily and commonly acquired, may explain the predominance of HSV-2.

A retrospective study of acute viral CNS infections in Northern Greece (May 2014–December 2016) analyzed 132 hospitalized patients using multiplex PCR and serology, identifying viral pathogens in 52 cases (39.4%) [33]. VZV accounted for 13.5% of infections, while HSV-1 and HSV-2 were included among other viral causes, further supporting VZV’s notable contribution to CNS infections in Greece.

Our study found more HSV-1 and HSV-2 cases in autumn and winter, though not statistically significant, consistent with other reports [2,4,14]. In contrast, Lee et al. observed a summer peak for HSV-1, also without significance [25].

Consistent with prior studies, our study confirmed previously published data where CSF profiles in α-herpesvirus-positive cases typically show lympho- or monocytic pleocytosis (>10 cells/mm^3^), elevated protein (>40 mg/dL), and normal glucose [2,4,6,8,15,19,20,25,26,32,34]. Parkes Smith et al. reported higher CSF protein and cell counts in HSV-2 (and to a lesser extent VZV) compared with HSV-1 [6], while other studies found no significant intervirus differences [5,11,15,19]. Lee et al. observed higher cell counts in VZV than HSV-1 (302 vs. 117 cells/mm^3^; *p* = 0.034) [25], and Ma et al. similarly found elevated cell counts and protein in VZV versus HSV cases (230 vs. 87 cells/mm^3^; 103.4 vs. 69.4 mg/dL; *p* ≈ 0.01) [28]. Rarely, normal CSF findings occur, as in two VZV-positive elderly patients in Arruti et al.’s study [2].

In this study, CT imaging had limited diagnostic value and was primarily used to ensure lumbar puncture safety by excluding contraindications such as mass effect or elevated intracranial pressure [2,15]. MRI proved more sensitive, especially in encephalitis, with HSV-1-and to a lesser extent VZV-producing characteristic inflammatory lesions with virus-specific anatomical patterns. These findings are consistent with previous reports [2,6,11,19,25,33].

This study has several limitations. It is retrospective, with incomplete records and data gaps, and was conducted at a single center with a limited number of positive cases, reducing statistical power. Data were unavailable concerning patients’ dietary habits, VZV vaccination status, and their comprehensive immunological profile. Despite the small sample, findings are consistent with larger studies using similar diagnostic criteria, supporting their validity. Additionally, overlapping clinical features between encephalitis and meningitis complicate case classification, preventing distinct analyses for each condition.

## 5. Conclusions

This retrospective study highlights regional epidemiological differences in herpesvirus CNS infections, influenced by factors such as geography, sample size, vaccination coverage, population genetics and timing of data collection as diagnostic practices evolve [3,7,8,13,30,32,33]. VZV is often the most detected virus, likely due to increased testing (even without rash or immunosuppression), enhanced sensitivity of molecular diagnostics [PCR, next-generation sequencing (NGS)], and greater clinical recognition of atypical VZV CNS presentations [2,4,6,15,19,25,28,30,32,33,34]. Thus, early detection through PCR testing is crucial for timely antiviral treatment when indicated, improving outcomes and reducing mortality.

## Figures and Tables

**Table 1:** Annual distribution of positive cases throughout the study period.

	2019	2020	2021	2022	2023	2024	Total
HSV-1	2	2	2	1	0	2	9
HSV-2	4	0	1	0	2	0	7
VZV	4	7	7	4	4	11	37
Total	10	9	10	5	6	13	53

**Table 2:** Immunological status in positive cases. CLL: Chronic Lymphocytic Leukemia; SLE: Systemic Lupus Erythematosus.

Immunological Status	Number of Cases
No data available	25
Immunocompetent	16
Hepatic cirrhosis	1
Malignancies	5
Myasthenia Gravis	1
CLL	1
Rheumatoid Arthritis	1
SLE	2
Diabetes Mellitus Type 2	1

**Table 3:** MRI findings.

	Positive MRI	Negative MRI	Total
HSV-1	6	1	7
HSV-2	1	3	4
VZV	9	11	20
Total	16	15	31

**Table 4:** White blood cell count, CSF glucose, and protein levels in all positive cases. The table presents the mean, median, extreme values, and range for each virus regarding these parameters.

Virus		Cells (per mm^3^)	Glucose (mg/dL)	Protein (mg/dL)
HSV-1	N	8	8	8
	Mean	43.25	64.13	67.94
	Median	26	59.50	53.05
	Minimum	1	52	34.5
	Maximum	124	95	122.7
HSV-2	N	6	6	6
	Mean	220.67	46.5	124.82
	Median	231	42	131.85
	Minimum	107	31	71
	Maximum	350	76	185
VZV	N	32	31	31
	Mean	312.38	59.55	97.67
	Median	125	56	85.2
	Minimum	1	39	34
	Maximum	1860	118	245.8

## Data Availability

The original contributions presented in this study are included in the article. Further inquiries can be directed to the corresponding author.

## Abbreviations

The following abbreviations are used in this manuscript:

AUTH	Aristotle University of Thessaloniki
CBC	Complete Blood Count
CLL	Chronic Lymphocytic Leukemia
CMV	Cytomegalovirus
CNS	Central Nervous System
CRP	C-reactive Protein
CT	Computed Tomography
CSF	Cerebro-Spinal Fluid
HSV	Herpes Simplex Virus
IC	Internal Control
MRI	Magnetic Resonance Imaging
NGS	Next-Generation Sequencing
PCR	Polymerase Chain Reaction
SLE	Systemic Lupus Erythematosus
VZV	Varicella Zoster Virus
WBC	White Blood Cell
